# Polyphosphoinositides suppress the adhesion of *Haemophilus influenzae *to pharyngeal cells

**DOI:** 10.1186/1476-511X-3-20

**Published:** 2004-09-03

**Authors:** Jim-Wen R Liu, Steve N Anderson, Jonathan A Meulbroek, Shie-Ming Hwang, Pradip Mukerji, Yung-Sheng Huang

**Affiliations:** 1 Ross Products Division, Abbott Laboratories, 625 Cleveland Ave., Columbus, OH 43216, USA; 2 Pharmaceutical Products Division, Abbott Laboratories, Abbott Park, Chicago, IL 60064-6110, USA

## Abstract

**Background:**

One of the primary causes of otitis media (OM), an inflammation of the middle ear, is the bacterium *Haemophilus influenzae *(HI). OM often occurs to young children, and is mostly treated with antibiotics. Due to concerns over bacterial resistance toward antibiotics, reliable prophylactic treatments such as administrating anti-adhesion agents are now viewed as viable alternatives.

**Results:**

The present study tested the feasibilty of using phosphoinositides as anti-adhesion agents against HI cells. Cells of non-typeable HI were radiolabeled with ^111- ^indium-oxine, pre-incubated with various individual phosphoinositides for 15 minutes at 37°C, and incubated with a monolayer of human pharynx carcinoma (DT 562) cells for 20 minutes at 37°C. The result showed that at 0.1 mg/mL dipalmitoylphosphatidylinositol-3,4-diphosphate (PI-3,4-PP) had the highest anti-adhesion activity, followed by phosphatidylinositol-3-phosphate (PI-3-P) and phosphatidylinositol-4-phosphate (PI-4-P). The anti-adhesion activity of PI-3,4-PP was dose-dependent ranging from 0.006 to 0.1 mg/mL. In addition, results from an *in vivo *study demonstrated that pre-incubation of HI cells with PI-3,4-PP at 1 mg/mL suppressed the growth of HI in nasopharynx of neonatal rats.

**Conclusions:**

These findings suggest that PI-3-P and PI-4-P and more so PI-3,4-PP may serve as prophylactic agents against HI adhesion and colonization.

## Background

Otitis media (OM) is an inflammation of the middle ear, often seen in children younger than six year of age. OM is caused by infection of nasopharyngeal cells by the bacterium *Haemophilus influenzae *(HI). Complications of OM include permanent hearing loss and perforation of the tympanic membrane.

Generally, OM is treated with antibiotics such as penicillin derivatives. However, in spite of the effectiveness of antibiotic prophylaxis, the increasing bacterial resistance to antibiotics has caused some concerns. This has prompted the development of anti-adhesive agents against HI infection [1]. Today, several anti-adhesive agents such as xylitol and oligosaccharides have been studied in clinical trials [2-5]. Human casein has been shown to have an inhibitory effect on the adhesion of HI to human respiratory tract epithelial cells [6], but the active factor(s) has not been characterized. Recently, we have discovered that certain rice flour extract inhibits HI adhesion [7]. Results from the preliminary purification process indicated that the active factor(s) in the rice flour extract was amphiphilic and structurally resemble to the phosphoinositides. To verify this view, we examined in this study the anti-adhesion activities of phosphoinositides against HI using the models of human pharynx carcinoma (DT 562) cells and neonatal rats.

## Results and Discussion

### Bioactivity of polyphosphoinositides

**Figure 1:**
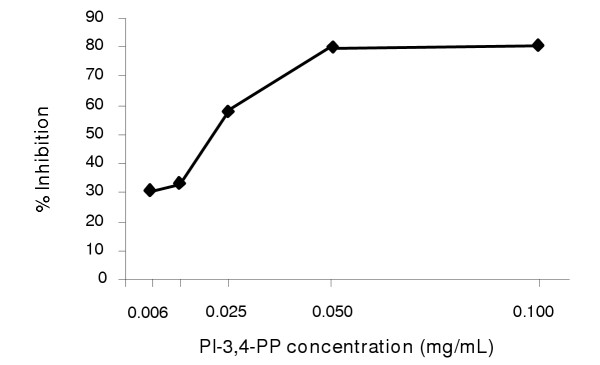
The dose response curve of PI-3,4-PP against the adhesion by *H. Influenzae*.

The attachment (adhesion) of bacteria to a mammal's nasopharynx area is believed to be the first stage of the bacterial infection, which can lead to OM and other disorders and diseases caused by HI. For this study, *in vitro *effect of various phosphoinositides on the attachment of HI to nasopharyx was evaluated. The activity was defined as the percent of inhibition of HI adhesion to human pharynx carcinoma cells as compared to the control. Results in Table 1 show that four phosphoinositides, i.e., Ins-1,2,6-PPP, Ins-1,3,4-PPP, GPI and PI at a concentration of 0.1 mg/mL exerted no effect on HI adhesion to the human pharynx cell cultures. On the other hand, PI-3-P and PI-4-P showed 24% inhibition of HI adhesion. More, PI-3,4-PP at the same concentration showed 74% inhibition against HI adhesion. A dose-dependent inhibition of HI adhesion by PI-3,4-PP was observed between 0.006 and 0.1 mg/mL, which exerted 31 to 81% inhibition (Figure 1).

**Table 1:** Bioactivities (% inhibition) of phosphoinositides against the adhesion by *Haemophilus influenzae*

Compound	% Inhibition	Dose (mg/mL)
Ins-1,2,6-PPP	6^a^	0.1
Ins-1,3,4-PPP	-1	0.1
GPI	-3	0.5
PI	-2	0.1
Pl-3-P	24	0.1
Pl-4-P	24	0.1
PI-3,4-PP	74	0.1

^a ^Within statistical error; thus no activity is presumed.

### In Vivo Activity of PI-3,4-PP

**Figure 2:**
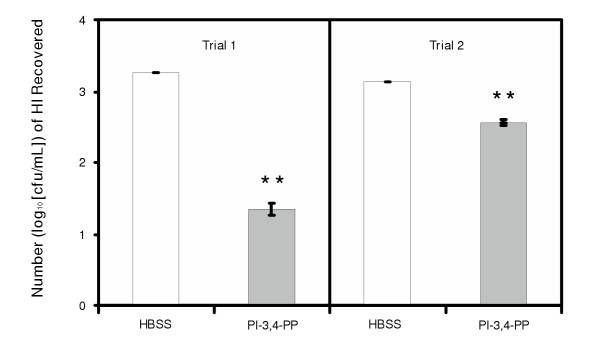
*In vivo *activity of 1 mg/mL of PI-3,4-PP against the adhesion by *H. influenzae*.

The inhibitory activity of PI-3,4-PP against the attachment of nontypeable HI was further demonstrated in two trials using a neonatal rat model. Figure 2 shows the average inoculum dose (cfu) and the average number (log_10 _[cfu/mL]) of HI recovered for a total of 10 rat pups for each treatment. In trial 1, at the inoculum dose of approximately 100 cfu/pup, the treated group (PI-3,4-PP) containing 1 mg/mL of PI-3,4-PP showed an 80-fold (1.9 logs) reduction in the number of bacteria recovered 24 hours post-inoculation as compared to the control group (HBSS). In trial 2, the rat pups were exposed to much higher doses (approximately 700 cfu/pup) of bacteria. The treated group (PI-3,4-PP), which was protected by the same level of PI-3,4-PP, showed a 4-fold (0.6 log) reduction in the number of bacteria recovered. The result demonstrates that PI-3,4-PP can inhibit the attachment and thus the growth of nontypeable HI in the nasopharynx of neonatal rats.

The exact mechanism for the anti-adhesion activity of the phosphoinositides is not known at this time. However, the anti-adhesion activity may be associated with the structures of the phosphoinositides. Molecules containing no phosphatidyl group such as Ins-1,2,6-P, Ins-1,3,4-P and GPI, exerted no effects on adhesion. PI, which contains a phosphatidyl group, also had no activity. In addition to the presence of a phosphatidyl group, phosphorylation of inositol seems to be required for the bioactivity. Since PI-3,4-PP had a higher anti-adhesion activity than PI-3-P and PI-4-P, it is possible that the presence of extra phosphate groups at the positions 3 and 4 of the inositol moiety may exert an even greater effect. Results from a preliminary study (Jeffrey Baxter, personal communication) showed the presence of multi-phosphate groups in a molecule (such as phytate, inositol phosphate, and phosvitin) were inhibitory to HI adhesion. On the other hand, results from our study showed that PI-3,4-PP was more active than phytate (a *myo*-inositol hexaphosphate), *myo*-inositol penta-and tetra-phosphates (data not shown). Taken together, these results suggests that the presence of both a phosphatidyl group and an additional phosphate group is essential for the high anti-adhesion activity of PI-3,4-PP as compared to PI-3-P and PI-4-P.

It is known that bacterial adhesion involves specific recognition of carbohydrate receptors by pathogen proteins [4]. This specificity is probably one of the main factors that dictate in which tissue that pathogen species can successfully colonize. Previously, the anti-adhesion effect of some oligosaccahrides has been attributed to their bindings to the specific binding sites of pathogen's proteins [4]. These oligosaccharides serve as decoys and occupy bacteria's carbohydrate-binding proteins, and thus reduce the binding of pathogens to the native carbohydrate in epithelial cell membrane. Similarly, polyphosphoinositides present in inner ear tissue and kidney [8,9] and other tissues have been postulated as *in vivo *receptor for aminoglycoside antibiotics [10]. It is possible that phosphorylated phosphatidylinositol such as PI-3-P, PI-4-P, and PI-3,4-PP may serve as decoys by occupying the binding sites in bacteria and prevent their attachment to the epithelial cells. It has also been reported that phosphatidylinositides can bind to mCD14, a cell-surface receptor on the membrane of monocytes and neutrophils [11]. Among those tested phosphatidylinositides, PI-3-P, PI-4-P, PI-3,4-PP, and PI-4,5-PP display the highest affinities for mCD14. Recently, non-typeable HI have been shown to adhere to human bronchial epithelial cells through the lipooligosaccharide (LOS) on the cell surface of the bacteria [12], and LOS further interacts with the platelet-activating factor (PAF) receptor to initiate host cell signal cascade and bacterial invasion [13]. It is then possible that phosphatidylinositides occupy the binding sites of the bronchial epithelial cells through binding to the membrane of these cells, thus reducing the number of binding sites available for HI attachment. By suppressing the binding of HI to the cell membrane, phosphatidylinositides prevents the replication of the bacteria.

## Conclusions

In conclusion, the PI-3,4-PP suppressed the adhesion of nontypeable HI to nasopharyngeal cells of neonatal rats, and thus preventing the replication of the bacteria in the animal. The results suggest that the phosphoinositides may be used to formulate pharmaceutical and nutritional compositions for prophylactic treatments of OM and other infections caused by HI.

## Materials and Methods

### Phosphoinositides

**Figure 3:**
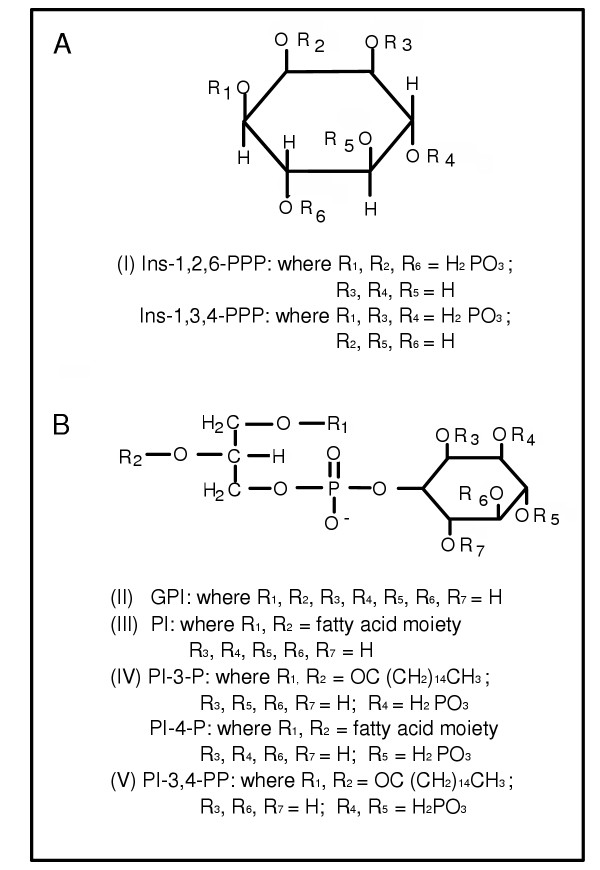
Structural formulas of the five (I through V) classes of phosphoinositides.

Five different classes (I through V) of phosphoinositides were used for this study (Figure 3). Class I included 1-D-*myo*-inositol-1,2,6-triphosphate sodium salt (Ins-1,2,6-PPP) and 1-D-*myo*-inositol-1,3,4-triphosphate sodium salt (Ins-1,3,4-PPP) (Figure 3A). Class II included 1-(α-glycerophosphoryl)-D-*myo*-inositol lithium salt (GPI), class III L-α-phosphatidylinositol ammonium salt (PI), class IV dipalmitoylphosphatidyl-inositol-3-phosphate ammonium salt (PI-3-P), and L-α-phosphatidylinositol-4-monophosphate sodium salt (PI-4-P), and class V dipalmitoylphosphatidylinositol-3,4-diphosphate ammonium salt (PI-3,4-PP) (Figure 3B). Ins-1,2,6-PPP, Ins-1,3,4-PPP, PI-3-P, and PI-3,4-PP were obtained from Matreya Inc. (Pleasant Gap, PA), GPI from Calbiochem-Novabiochem Corp. (La Jolla, CA), and PI and PI-4-P from Sigma Chemical Co. (St. Louis, MO). All chemicals are reagent grade with purity greater than 99%.

### Cell Cultures

The Detroit 562 human pharynx carcinoma cell line (DT 562) was obtained from the American Culture Type Collection. The DT 562 cells were seeded into Costar 96-well plates (Corning Life Science, Acton, MA) at a density of 20,000 to 25,000 cells per well, and cultured in Dulbeco's modified Eagle Medium (GIBCO, Grand Island, NY) supplemented with 10% fetal bovine serum (FBS) (Hyclone, Logan, UT). The plates were incubated in a humidified atmosphere of 95% air: 5% CO_2 _at 37°C until reaching at least 90% confluency. Plates were washed three times with 20 mL of Hanks Balanced Saline Solution (HBSS) (Sigma Chemical Company, St. Louis, MO) to remove serum proteins.

### Radiolabeling of HI bacteria

For the adhesion study, an HI nontypeable bacterial strain was used. The HI isolated from the middle ear of an infected child was a gift from Dr. Lauren Bakaletz of The Ohio State University, Columbus, Ohio. HI was streaked onto Chocolate agar plates (Becto Dickinson Diagnostic Instrument System, Sparks, MD) from frozen aliquots of a low passage number. The plates were then incubated at 37°C in a humidified atmosphere of 95% air: 5% CO_2 _for 18 hours. Bacteria were then harvested in phosphate buffered solution (PBS) supplemented with 0.05% bovine serum albumin (BSA) (Miles Inc., Kankakee, Ill.). After centrifugation, the cell pellets were resuspended in a volume of PBS/BSA yielding an optical density of 2.4 at a wavelength of 660 nm. The bacteria were then radiolabelled with ^111-^Indium-oxine (^111- ^In), a high energy, short-lived tracer. Fifty μCi of ^111-^In solution was added to 2.5 mL of the bacterial suspension and incubated for 20 minutes at 37°C. The radiolabeled bacteria were then washed two times with 10 mL HBSS and unbound ^111- ^In were removed by centrifugation. The bacteria pellets were then resuspended in 5 mL HBSS supplemented with 30 mM 2-hydroxyethyl-piperazine-N'-2-ethane sulfonic acid buffer (Life Technologies, Calsbad, CA).

### Adhesion Quantitation

Prior to adhesion test, aliquots (25 μL) of the ^111- ^In-labeled bacterial suspension were pre-incubated with 25 μL of the test chemical (containing various phosphoinositides) in a Costar polypropylene 96-well plate for 15 minutes at 37°C to allow binding of the test agent to the HI. For adhesion quantitation, aliquots (25 μL) of the pre-incubated mixture were pipetted into the wells of an assay plate containing the DT 562 human pharynx carcinoma cells. The assay plate was incubated for about 15 to 20 minutes at 37°C to allow adhesion of the bacteria to the cell monolayer. Nonadherent bacteria were removed by washing the plate three times with HBSS. The cell monolayer and the adhering of the HI cells were disrupted by the addition of 100 μL of 0.05 N sodium hydroxide. The contents of each well were placed in Cobra polypropylene tubes and the radioactivity counted on a Cobra Gamma Counter (Packard Instrument Co., Meriden, CT). After calibration of the background, the average radiation count of four replicates (per sample) was calculated. The percents of inhibition of bacterial adhesion, as compared to bacterial attachment in control wells containing no test chemical were then calculated.

### In Vivo Activity

A neonatal rat model [14,15] was modified for testing the *in vivo *activity of PI-3,4-PP against nontypeable HI. Prior to the test, overnight cultures of nontypeable HI were prepared, washed twice and diluted with HBSS to obtain a bacterial suspension of less than 10^5 ^colony-forming-units (cfu) per mL. Three different test chemicals were prepared for this test: (i) HI + PI-3,4-PP, an aliquot (0.5 mL) of PI-3,4-PP (2 mg/mL in HBSS) mixed with 0.5 mL of the diluted bacterial suspension (5 × 10^4 ^cfu/mL) and incubated for one hour at 37°C; (ii) HI + HBSS, This bacterial control prepared by mixing and incubating 0.5 mL HBSS with 0.5 mL of the diluted bacterial suspension; and (iii) HBSS, the solvent blank prepared by incubating 1 mL of HBSS under the same conditions. A 10 μL of the test material was used to inoculate 24-hour-old or younger Sprague Dawley rats (Charles River, Portage, MI) by intranasal administration. Twenty-four hours after the inoculation, samples of nasopharyngeal fluid were collected by the slow instillation of 25 μL of HBSS into the left naris, and the initial 10-μL discharge from the right naris was collected for plate count. This procedure insured that the fluid had passed through the nasopharynx. The nasal wash was then spread (or diluted and then spread), onto Chocolate agar plates. The plates were incubated at 37°C overnight and counted for the number of cfu's, an indicator of the number of viable bacteria.

## Authors' contributions

YSH conceived of the study. JWL screened and identified the phosphoinositides, and drafted the manuscript. SNA carried out cell adhesion assay. JM carried out assay for in vivo activity. SMH and PM participated in experiment coordination.
